# A cross-sectional study on public health nurses' disaster competencies and influencing factors during the COVID-19 pandemic in Korea

**DOI:** 10.1186/s12889-022-13091-2

**Published:** 2022-04-13

**Authors:** Eunjoo Hong, Aeri Jung, Kyungmi Woo

**Affiliations:** 1 College of Nursing, Seoul National University, 103 Daehak-ro, Jongno-gu, Seoul, Republic of Korea; 2 College of Nursing, Eulji University, 712 Dongil-ro, Uijeongbu-si, Gyeonggi-do Republic of Korea

**Keywords:** COVID-19, Disaster competencies, Emerging infectious diseases, Public health nurses, Self-determination theory

## Abstract

Public health nurses are performing various roles during the COVID-19 pandemic: counseling, surveillance, specimen collection, epidemiological investigation, education, and vaccination. This study investigated their disaster competencies in the context of emerging infectious diseases, and identified their influencing factors based on Deci and Ryan’s self-determination theory. A convenience sample of 242 was selected from public health nurses working in a metropolitan city of South Korea. Data were collected using a structured questionnaire and analyzed using descriptive statistics, t-test, one-way ANOVA, Pearson’s correlation, and multiple regression analysis using the SPSS Statistics ver. 23.0. Results showed that the significant factors influencing disaster competencies included “willingness to respond to a disaster,” “preventive behavior,” “experience of receiving education on emerging infectious diseases response,” “public health center experience,” “job satisfaction,” and “education.” This regression model explained 33.2% of the variance in disaster competencies. “Willingness to respond to a disaster” was the strongest factor affecting disaster competencies. Based on these results, it is concluded that interventions to improve disaster competencies and psychological well-being of public health nurses are needed. Additionally, strategies such as creating a supportive work environment, deploying experienced nurses primarily on the front line, and reducing the tasks of permanent public health nurses should be implemented.

**Supplementary Information:**

The online version contains supplementary material available at 10.1186/s12889-022-13091-2.

## Background

Coronavirus disease 2019 (COVID-19), caused by the SARS-CoV-2 pathogen, is generally transmitted through respiratory droplets and has an incubation period of five to seven days. Infected individuals may be asymptomatic or develop serious symptoms including fever, cough, difficulty breathing, chills, headache, sore throat, and loss of taste or smell [1]. South Korea’s crisis alert level for COVID-19 has been raised to “serious”, as the number of confirmed cases and deaths continue to rise worldwide [2].

Infectious diseases are considered social disasters just as earthquakes, floods, and typhoons are considered natural disasters. Disaster competencies are crucial because an immediate and effective response to disasters directly impacts the life and safety of people. The International Council of Nurses suggests imparting disaster nursing competencies to nurses, who form the core of healthcare professionals and serve in a variety of important disaster response roles, such as initial response, severity classification, direct patient care, site management, and providing information, education, and psychological counseling [3, 4].

Given the explosive increase in COVID-19 cases in Korea, all public health nurses are now performing a dual role. Generally, they serve the roles of care provider, educator, counselor, source requester, case manager, cooperator, case finder, instructor, change agent, policy spokesperson, and social marketer [5]. However, now public health centers have become primary screening clinics in which public health nurses play additional roles, such as emergency planning, screening, specimen collection, surveillance, and epidemiological investigation [6, 7]. These nurses frequently contact undiagnosed people, thus exposing themselves to a high risk of infection, which causes mental stress, fatigue, stigma, and burnout [8–10].

Psychological problems of healthcare professionals are associated with their professional competencies, which are also directly related to the quality of nursing care and medical service. Therefore, managing the work environment of public health nurses is critical [10, 11]. Public health nurses are an important line of defense for the society, and therefore, their psychological health must be protected and their disaster competencies augmented for an effective response to COVID-19 [12]. This will entail the provision of a lot of support, which necessitates extensive research on the topic [13].

The factors that affect the competencies of public health nurses include age, employment type, job stress, emotional labor, and quality of professional life [14]. However, little research has been conducted to examine the correlation between these factors and disaster competencies of nurses. Consequently, there is a lack of awareness regarding how to equip nurses with disaster competencies, apart from insufficient opportunities for receiving disaster nursing education [15, 16].

Public health nurses are today facing an increasing risk of burnout due to a prolonged response to COVID-19, exacerbated by shortage of trained workforce; therefore, a system of support and communication is now indispensable [17]. Identifying the factors that influence the professional competencies—including disaster management competencies—of public health nurses, who serve on the front line of the battle against emerging infectious diseases, such as COVID-19, will be helpful in developing and implementing educational programs and policies for effective workforce management and enhancement of professional competencies of public health nurses.

This study aimed to investigate the level of disaster competencies of public health nurses in the context of emerging infectious diseases, and identify influencing factors of disaster competencies based on the self-determination theory of Deci and Ryan [18].

**Fig. 1:**
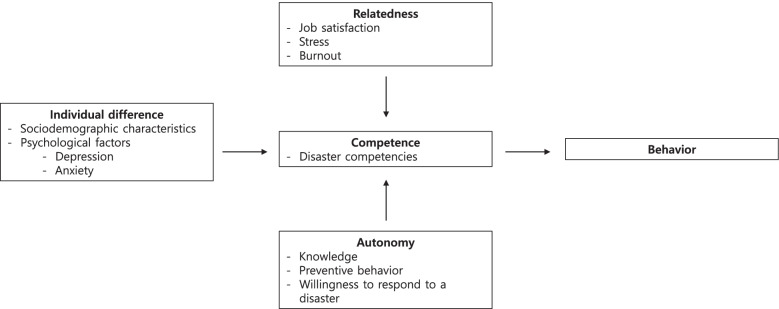
Research framework based on the self-determination theory of Deci and Ryan (2013). (We revised variable names such as Burnout and willingness to respond to a disaster as advised.)

This study applied the self-determination theory developed by Deci and Ryan [18] to public health nurses and constructed a research framework based on their disaster competencies in the context of emerging infectious diseases, and other relevant factors from three perspectives: autonomy, competence, and relatedness (Fig. 1). This theory, which emphasizes the importance of intrinsic motivation when humans decide to act or behave, is valid for studying healthcare professionals’ disaster competencies and related factors by associating them with their intrinsic motivation in the context of a social disaster, such as COVID-19.

The four factors of individual differences, autonomy, competence, and relatedness—the last three from Deci and Ryan’s self-determination theory—become the foundation for the adjusted behavior, emotion, or cognition of participants. More specifically, individual differences include sociodemographic characteristics and psychological factors (depression, anxiety); autonomy includes knowledge, preventive behavior, and the willingness to respond to a disaster situation, as it may influence behavior depending on the autonomous intention of participants; competence includes disaster competencies, as it is related to capability, skill, and talent of participants; and relatedness includes job satisfaction, stress, and burnout, which are affected by external factors. In this study, the output of consistent interaction between these four factors is set as disaster preparedness behavior according to the self-determination theory.

## Methods

### Study design

This research is a descriptive cross-sectional study designed to identify the disaster competency level of public health nurses in Busan, Gyeongsangnam-do Province in the context of emerging infectious diseases, and their influencing factors, based on Deci and Ryan’s self-determination theory.

### Participants

The participants of this study included public health nurses working at a public health center for at least six months in the Busan-Gyeongnam area. They were recruited through convenience sampling among those who satisfied the selection criteria and wished to voluntarily participate in the study. The selection criteria included:

1. Adults aged 18 or above
2. Able to read and understand Korean
3. Public health nurses working in public health centers (including health center branch, community health center, district service center, and community health promotion center) for at least six months
4. Able to understand the research purpose
5. Voluntarily participation in the study

We selected only those public health nurses who had at least six months of experience working in public health centers because of the possibility of receiving incomplete questionnaires from individuals with less than six months of experience, as they may not possess sufficient understanding of tasks at public health centers.

The number of samples needed for the study was calculated to be 222, using G*Power 3.1.9.7 with an effect size of 0.15, 20 predictors, a significance level of 0.05, and statistical power (1-β) of 0.95. A total of 242 individuals were surveyed, considering the possibility of receiving incomplete questionnaires. A total of 242 individuals were chosen as final participants with 100.0% response rate.

### Instrument

All instruments were used after obtaining approval from one of the authors who could be contacted for each instrument.

### Depression

The severity of depression was measured using the patient health questionnaire (PHQ)-8 excluding one item on suicidal ideation from the Korean version of the instrument [19], which has been translated from the PHQ-9 developed by Spitzer et al. [20]. Each of the eight questions was scored based on a 4-point Likert scale. The range of total scores was 0 to 24 points and higher scores indicated higher severity of depression. Cronbach’s α for the reliability of the Korean version of the PHQ-8 tool was 0.88 [21], and 0.911 in this study.

### Anxiety

The participants’ anxiety was measured using the Korean translated-version of the Generalized Anxiety Disorder-7 (GAD-7) tool developed by Spitzer et al. [22]. Each of the seven questions was scored based on a 4-point Likert scale. The range of total scores was 0 to 21 points and higher scores indicated higher severity of anxiety. Cronbach’s α for the reliability of the Korean version of the GAD-7 tool was 0.924 [23], and 0.928 in this study.

### Job satisfaction

The level of job satisfaction was measured using four items related to job satisfaction in the Korean version of the Copenhagen Psychosocial Questionnaire version II Scale (COPSOQ-K), which has been adapted from the COPSOQ II [24]. Each of the four questions was scored based on a 4-point Likert scale. The range of total scores was 4 to 16 points and higher scores indicated better job satisfaction. Cronbach’s α for the reliability of the COPSOQ-K was 0.78 [25], and 0.836 in this study.

### Stress

The level of stress was measured using the Korean translation of the Brief Encounter Psychosocial Instrument (BEPSI-K) developed by Frank and Zyznaski [26]. A total of five questions were scored based on a 5-point Likert scale. The total score ranged from 5 to 25, where it was divided by 5 to calculate the average value for evaluating the level of stress. Cronbach’s α for the reliability of the Korean version of the validity study [27] was 0.80, and 0.834 in this study.

### Burnout

The Copenhagen Burnout Inventory (CBI) developed by Kristensen et al. [28] and translated into Korean [29] was used to measure the severity of burnout. Each of the 19 questions was scored based on a 5-point Likert scale. Higher scores indicated higher severity of burnout. Cronbach’s α for the reliability in a previous study, which measured the severity of burnout among newly graduated nurses and preceptors, were 0.924 [29], and 0.957 in this study.

### Disaster competencies

Disaster competencies were measured using the Korean version of the Disaster Preparedness Evaluation Tool for nurses (DPET-K) [11], which has been adapted from the DPET developed by Bond and Tichy (2007) for evaluating the knowledge and skills related to disaster management among nurses [30]. This instrument covers nursing competencies required in three disaster management stages: the prevention stage consisting of three domains including disaster education training, disaster knowledge and information, and bioterrorism and emergency response; the mitigation stage including the disaster response domain; and the recovery stage including the disaster evaluation domain. A total of 28 items were scored based on a 6-point Likert scale where higher scores indicated a higher level of disaster competencies. Cronbach’s alpha of DPET-K and in this study were 0.954 [11] and 0.962, respectively.

### Knowledge

The level of knowledge was measured by revising and improving the COVID-19 knowledge instrument used in a study related to COVID-19 [31] conducted among nurses in general hospitals, based on the COVID-19 response guidelines no. 9–4 [32], to be more appropriate for the current situation. The content validity index was verified during revision and improvement by a group of experts consisting of three professors of nursing, two working-level staff responding to disasters at public health centers, and two individuals with the experience of developing disaster instruments; the content validity index (CVI) was 1.00. The participants were instructed to answer “yes” or “no” for a total of 20 items, and the score ranged from 0 to 20. Higher scores indicated a higher level of knowledge. The reliability of the original instrument had the Kuder-Richardson 20 (KR-20) of 0.15; Cronbach’s alpha in this study was 0.378.

### Preventive behavior

Preventive behavior was measured using the instrument that has been revised and supplemented from the preventive behavior instrument used in a COVID-19 study conducted among the general public [33]. The content validity index was verified during revision and improvement by a group of experts consisting of three professors of nursing, two working-level staff responding to disasters at public health centers, and two individuals with the experience of developing disaster instruments; CVI was 1.00. A total of 14 items were scored based on a 4-point Likert scale where higher scores indicated a higher level of compliance with preventive behavior. Cronbach’s alpha in the original instrument and in this study were 0.875 and 0.869, respectively.

### Willingness to respond to a disaster

Willingness to respond to a disaster was measured by revising and supplementing the Korean version [34] of the instrument originally developed by Qureshi et al. [32], to make it more appropriate for the study region. The original instrument consisted of seven scenarios including snow storm, smallpox, chemical terrorism, explosion, wild fire-related asthma attack, radioactive terrorism, and severe acute respiratory syndrome. However, the instrument has been revised according to the current situation and regional characteristics of Korea, and includes eight scenarios: snow storm, flood, chemical terrorism, explosion, earthquake, landslide, radioactive terrorism, and emerging infectious diseases. The participants were instructed to choose among “willing to work as disaster response workforce (1 point),” “not willing to work as disaster response workforce (0 point),” or “do not know (0 point);” higher scores indicated a higher degree of willingness to respond to a disaster. The content validity index was verified during revision and improvement by a group of experts consisting of three professors of nursing, two working-level staff responding to disasters at public health centers, and two individuals with the experience of developing disaster instruments; CVI was 0.88. The reliability in a previous study [34] had the KR-20 of 0.90; Cronbach’s alpha in this study was 0.941.

### Data collection

The data collection period of this study was from March 27 to April 6, 2021, and the participants were instructed to complete a self-report online survey on Google. After obtaining the approval of the Institutional Review Board (IRB), the researcher visited public health centers in the Busan-Gyeongnam region and explained the purpose and procedure of the study to recruit study participants. Furthermore, the recruitment post was shared through the messenger service used by the staff at public health centers to induce individuals to contact the researcher or access the online survey if they wished to participate in the study.

### Statistical procedures and analysis

The collected data were analyzed using SPSS ver. 23.0. General characteristics of the participants and the level of each variable were analyzed using descriptive statistics, such as frequency, percentage, mean, and standard deviation. The reliability of the instrument was analyzed using Cronbach’s α. The T-test and ANOVA were performed to examine the difference in each variable with respect to general characteristics. Pearson's correlation coefficient was used to evaluate the correlation between the variables. The multiple regression analysis was performed to examine which factors influence disaster competencies. Every categorical variable was converted to a dummy variable when inputting variables. The stepwise regression analysis was performed after removing one outlier for which the absolute value of a standardized residual was greater than 3 during case-wise diagnostics. For verifying normality of residuals, homoscedasticity, and linearity—the basic assumptions of the regression analysis—histogram of the standardized residual and the normal probability plot of the standardized residual, and the scatter plot were examined. All statistical tests were two-sided, with *p* < 0.05 considered statistically significant.

### Ethical considerations

This study protocols for the collection and analyses of the survey data were approved by the Institutional Review Board of Seoul National University (No: 2102/001–008). The study purpose, option to voluntarily withdraw from the study, and anonymity were explained on the first page of the online survey, and the participants’ informed consent was obtained. A mobile gift card of 10,000 won was presented to the participants who completed the online survey as a token of appreciation and to increase the reliability of the survey responses. All procedures were performed in accordance with the relevant guidelines and regulations. Permission to use the study instruments was obtained from the author through e-mail or over the phone prior to data collection.

## Results

### Sample description

The sample description of the participants is provided in Table 1. A total of 97.5% (*n* = 236) of the participants were female, and the average age was 37.24 years (± 9.78). A total of 71.9% (*n* = 174) of the participants possessed a bachelor’s degree in nursing. The average total work experience as a nurse was 10.2 years (± 8.46). The average duration of work experience at public health centers was 6.6 years (± 7.81). Regarding the experience related to emerging infectious diseases, 65.7% (*n* = 159) had received education on response to emerging infectious diseases, including COVID-19, while 90.1% (*n* = 218) responded they had experience working at COVID-19 screening clinics.

**Table 1:** Sociodemographic Characteristics. (*N* = 242)

Characteristics	Categories	N (%) or M ± SD
Gender	Female	236(97.5)
	Male	6(2.5)
Age		37.24 ± 9.78
	≦29 years old	64(26.4)
	30–39 years old	91(37.6)
	40–49 years old	51(21.1)
	50–59 years old	33(13.6)
	≧60 years old	3(1.2)
Education	Associate’s degree in nursing	52(21.5)
	Bachelor’s degree in nursing	174(71.9)
	Pursuing/Master’s degree in nursing	16(6.6)
Religion	Christian	40(16.5)
	Catholic	23(9.5)
	Buddhist	45(18.6)
	No Religion	129(53.3)
	Others	5(2.1)
Marital status	Single	107(44.2)
	Married	131(54.1)
	Other	4(1.7)
Child	One or more	102(42.1)
	None	140(57.9)
First Child’s Age		15.64 ± 9.51
	≦5 years old	14(5.8)
	6–12 years old	34(14.0)
	13–18 years old	15(6.2)
	≧19 years old	39(16.1)
Total work experience as a nurse		10.2 ± 8.46
	< 1 year	12(5.0)
	1–4 years	56(23.1)
	5–9 years	73(30.2)
	10–19 years	67(27.7)
	20–29 years	19(7.9)
	≧30 years	15(6.2)
Public health nurse experience		6.6 ± 7.81
	< 1 year	40(16.5)
	1–4 years	101(41.7)
	5–9 years	48(19.8)
	10–19 years	36(14.9)
	20–29 years	6(2.5)
	≧30 years	11(4.5)
Employment type	Permanent	174(71.9)
	Non-fixed term	32(13.2)
	Fixed-term	36(14.9)
Work location	Busan	139(57.4)
	Changwon	12(5.0)
	Gimhae	18(7.4)
	Yangsan	15(6.2)
	Others	58(24.0)
Place of employment	Public health center	209(86.4)
	Health center branch	6(2.5)
	Community health center	5(2.1)
	District service center	7(2.9)
	Community health promotion center	7(2.9)
	Others	8(3.3)
Work department (if working at public health centers)	Infectious disease related	52(21.5)
	Non-infectious disease related	157(64.9)
Experience of receiving education on emerging infectious diseases response	Yes	159(65.7)
	No	83(34.3)
Experience of working at COVID-19 screening clinics	Yes	218(90.1)
	No	24(9.9)

COVID-19: coronavirus disease 2019

### Descriptive statistics of the study variables

**Fig. 2:**
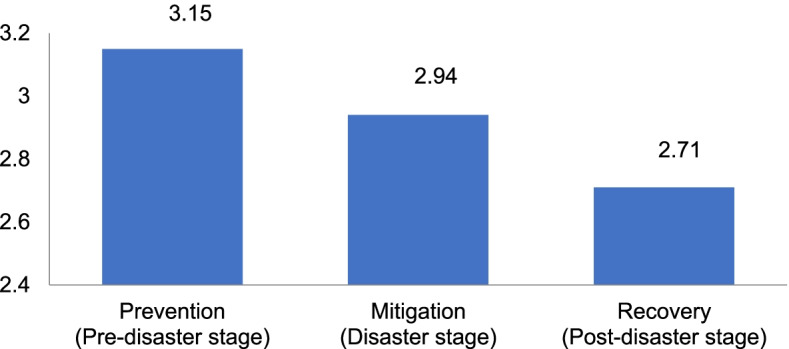
Disaster competencies average score. Columns indicate the average scores for each stage

**Fig. 3:**
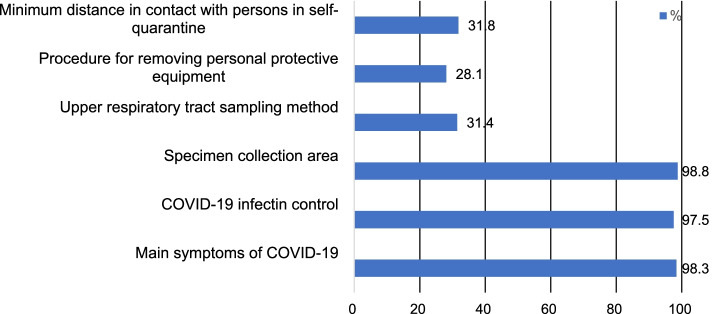
Percentage of correct answers for COVID-19 knowledge questions (top 3 and bottom 3). Columns indicate the proportions of participants who answered correctly

**Fig. 4:**
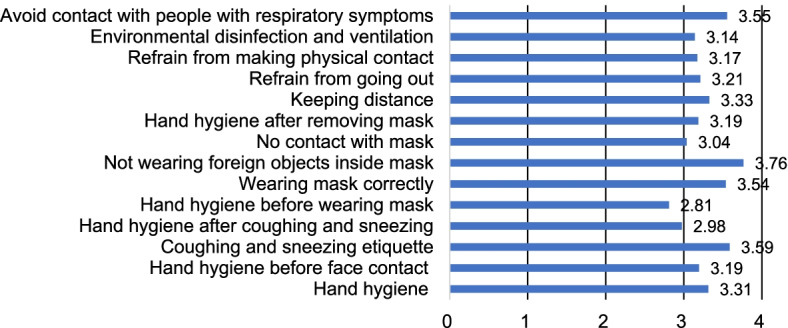
Preventive behavior average score. Columns indicate the average scores for each question

**Fig. 5:**
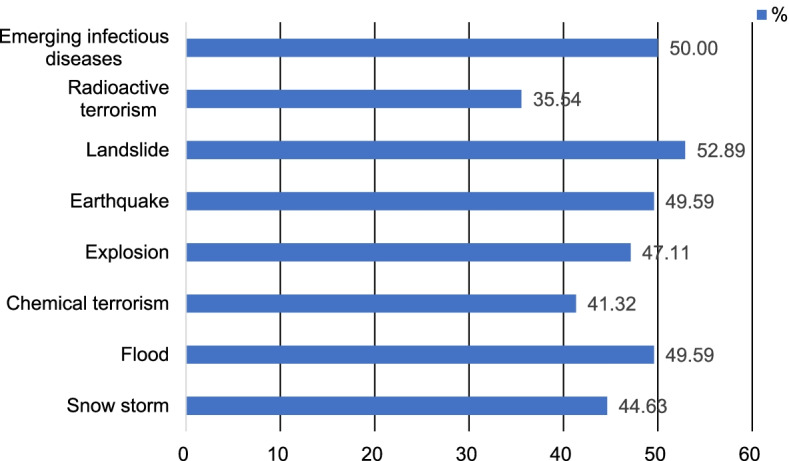
Willingness to respond to a disaster for each scenario. Columns indicate the proportions of participants who reported their willingness to work as disaster response workforce

The results of descriptive statistics of the study variables are presented in Table 2. The participants’ average score of depression was 7.51 ± 5.67 out of 24 points, and 32.23% (*n* = 78) of them had a score of at least 10 points—the PHQ-8 cut-off value. Their average score of anxiety was 5.11 ± 4.93 out of 21 points, and 17.36% (*n* = 42) of them had a score of at least 10 points—the GAD-7cut-off value. A total of 17.36%, or 42 participants, belonged to the anxiety group (GAD ≧ 10), 11.57% belonged to the moderate anxiety group (10 ≦ GAD < 15), and 5.79% belonged to the severe anxiety group (GAD ≧ 15). The average score of job satisfaction was 9.92 ± 2.33 out of 16 points. The stress level was evaluated by dividing the total score by 5, and the average score was 2.14 ± 0.62. A total of 32.23% (*n* = 78) of the participants obtained a score of 2.4 or higher, which is the cut-off value of BEPSI. The average score of burnout was 53.48 ± 16.16 out of 95 points. The ratio of moderate-to-severe burnout of 50 points or higher was 51.65%. The average score of disaster competencies was 84.08 ± 24.74 out of 168 points. Among the various stages of a disaster, prevention (pre-disaster stage) (M = 3.15) was the highest, followed by mitigation (disaster stage) (M = 2.94), and recovery (post-disaster stage) (M = 2.71) as shown in Fig. 2. The average score of knowledge related to COVID-19 was 14.08 ± 2.15 out of 20 points. The item with the highest rate of correct answer was “specimen collection for COVID-19 testing must be performed at screening clinics or in isolated spaces at medical institutions” (Fig. 3). The average score of preventive behavior was 45.81 ± 5.44 out of 56 points. The item, “do not place towel or tissue inside a mask” had the highest average score (Fig. 4). The average score of willingness to respond to a disaster was 3.71 ± 3.34 out of 8 points. The number of participants who responded that they were willing to work as disaster response workforce was analyzed according to disaster type (Fig. 5).

**Table 2:** Descriptive Statistics of Variables. (*N* = 242)

Variables	M ± SD	Possible range
Depression	7.51 ± 5.67	0–24
Anxiety	5.11 ± 4.93	0–21
Job Satisfaction	9.92 ± 2.33	4–16
Stress	2.14 ± 0.62	1–5
Burnout	53.48 ± 16.16	19–95
Disaster Competencies	84.08 ± 24.74	28–168
Knowledge	14.08 ± 2.15	0–20
Preventive Behavior	45.81 ± 5.44	14–56
Willingness to Respond to a Disaster	3.71 ± 3.34	0–8

### The differences in study variables according to general characteristics

The differences in depression, anxiety, job satisfaction, stress, burnout, disaster competencies, knowledge, preventive behavior, and willingness to respond to a disaster according to general characteristics among the participants are as follows.

### Depression

Depression had a statistically significant difference according to employment type (*p* < 0.001).

### Anxiety

Anxiety had a statistically significant difference according to age (*p* = 0.033), religion (*p* = 0.019), employment type (*p* < 0.001), and experience of receiving education on emerging infectious diseases response (*p* = 0.003).

### Job satisfaction

Job satisfaction had a statistically significant difference according to age (*p* < 0.001), education (*p* < 0.001), marital status (*p* = 0.005), total work experience as a nurse (*p* = 0.007), public health center experience (*p* = 0.022), employment type (*p* < 0.001), experience of receiving education on emerging infectious diseases response (*p* = 0.017), and experience of working at COVID-19 screening clinics (*p* = 0.004).

### Stress

Stress had a statistically significant difference according to employment type (*p* = 0.026).

### Burnout

Burnout had a statistically significant difference according to age (*p* < 0.001), education (*p* = 0.004), marital status (*p* = 0.025), number of children (*p* < 0.001), age of child(ren)(*p* = 0.005), total work experience as a nurse (*p* = 0.003), public health center experience (*p* = 0.031), employment type (*p* < 0.001), work department (*p* = 0.004), experience of receiving education on emerging infectious diseases response (*p* = 0.001), and experience of working at COVID-19 screening clinics (*p* = 0.002).

### Disaster competencies

Disaster competencies had a statistically significant difference according to age (*p* = 0.005), education (*p* = 0.006), number of child(ren) (*p* = 0.019), total work experience as a nurse (*p* = 0.002), public health center experience (*p* = 0.002), and experience of receiving education on emerging infectious diseases response (*p* < 0.001).

### Knowledge

The level of knowledge of COVID-19 did not vary according to the general characteristics.

### Preventive behavior

Preventive behavior had a statistically significant difference according to age (*p* = 0.033), child’s age (*p* = 0.034), and employment type (*p* = 0.026).

### Willingness to respond to a disaster

Willingness to respond to a disaster had a statistically significant difference according to child’s age (*p* = 0.026) and experience of receiving education on emerging infectious diseases response (*p* = 0.002).

### Correlation between variables

The correlations between the variables are presented in Table 3. Disaster competencies had a statistically positive correlation with age (*r* = 0.230, *p* < 0.001), total work experience as a nurse (*r* = 0.226, *p* < 0.001), public health center experience (*r* = 0.256, *p* < 0.001), job satisfaction (*r* = 0.228, *p* < 0.001), preventive behavior (*r* = 0.312, *p* < 0.001), and willingness to respond to a disaster (*r* = 0.363, *p* < 0.001), but a statistically negative correlation with depression (*r* = -0.160, *p* = 0.012), stress (*r* = -0.172, *p* = 0.007), and burnout (*r* = -0.209, *p* = 0.001). Specifically, participants’ disaster competencies tended to be higher when their age, total work experience, public health center experience, and job satisfaction were higher; disaster competencies were lower when their levels of depression, stress, and burnout were higher. Contrarily, the first child’s age (*r* = 0.152, *p* = 0.126), anxiety (*r* = -0.118, *p* = 0.066), and the level of knowledge (*r* = -0.033, *p* = 0.607) did not have a significant correlation with disaster competencies.

**Table 3:** Correlation among the Research Variables. (*N* = 242)

Variables	1	2	3	4	5	6	7	8	9	10	11	12	13
1. Age	1	.882^b^	.782^b^	.676^b^	-.153^a^	-.147^a^	.223^b^	-.094	-.338^b^	.230^b^	.072	.140^a^	.122
2. First Child’s Age		1	.473^b^	.476^b^	-.155	-.195^a^	.134	-.189	-.390^b^	.152	.161	.083	.260^b^
3. Total Work Experience as a Nurse			1	.773^b^	-.124	-.129^a^	.194^b^	-.097	-.278^b^	.226^b^	.056	.092	.145^a^
4. Public Health Nurse Experience				1	-.049	-.061	.116	-.014	-.155^a^	.256^b^	.050	.124	.059
5. Depression					1	.824^b^	-.398^b^	.612^b^	.695^b^	-.160*	-.001	-.097	-.211^b^
6. Anxiety						1	-.415^b^	.651^b^	.709^b^	-.118	-.070	-.099	-.154^a^
7. Job Satisfaction							1	-.376^b^	-.579^b^	.288^b^	.040	.055	.317^b^
8. Stress								1	.635^b^	-.172^b^	-.031	-.104	-.161^a^
9. Burnout									1	-.209^b^	-.019	-.133^a^	-.224^b^
10. Disaster Competencies										1	-.033	.312^b^	.363^b^
11. Knowledge											1	-.037	.088
12. Preventive Behavior												1	.062
13. Willingness to Respond to a Disaster													1

^a^Correlation is significant at 0.05 (2-tailed)

^b^Correlation is significant at 0.01(2-tailed)

*P* < 0.05 is statistically significant

### Influencing factors of public health nurses’ disaster competencies

For examining the influencing factors of the participants’ disaster competencies, the variables that had a statistically significant difference, such as age, educational level, number of children, total work experience, public health center experience, and the experience of receiving education on emerging infectious diseases response, were entered. Moreover, the variables, such as depression, job satisfaction, stress, burnout, preventive behavior, and willingness to respond to a disaster, which had a significantly positive correlation with disaster competencies, were entered. The normality of residuals and homoscedasticity were examined through a histogram; the normal distribution of the data was also confirmed as the residual approached the straight line at 45° in the normal probability plot. Linearity and homoscedasticity were also confirmed in the scatter plot of residual as the distribution of residual was fairly even around 0. The Durbin-Watson statistic was 1.915, which is nearly twice the reference value, thus confirming no issue with autocorrelation. The absolute value of Pearson’s correlation coefficient of the independent variables included in the regression analysis ranged between 0.141 and 0.378, which is less than 0.8, thus confirming that all variables were independent. The tolerance value ranged between 0.867 and 0.976, which is greater than 0.1; there was no issue with multicollinearity as the variance inflation factor was not greater than 10 for the variables included in all the models. The range of Cook’s distance did not exceed the absolute value of 1, thus confirming that there are no outliers.

The regression analysis results showed that the regression model was significant (F = 20.841, *p* < 0.001), and the adjusted R^2^, which represents the explanatory power of the model, was 0.332. The factor with the greatest influence on disaster competencies was willingness to respond to a disaster (β = 0.267, *p* =  < 0.001), followed by preventive behavior (β = 0.256, *p* =  < 0.001), experience of receiving education on emerging infectious diseases response(β = 0.194, *p* =  < 0.001), public health center experience (β = 0.166, *p* = 0.002), job satisfaction (β = 0.148, *p* = 0.010), and educational level (pursuing or possessing a master’s degree) (β = 0.141, *p* = 0.009) (Table 4).

**Table 4:** Multiple Regression Analysis for Disaster Competencies of Public Health Nurses in Korea. (*N* = 242)

Variables	B	SE	β	t	p
(Constant)	-2.647	12.085		-.219	.827
Public health nurse experience	.043	.014	.166	3.061	.002
Education^a^ (Pursuing/Master’s degree in nursing)	13.891	5.282	.141	2.630	.009
Experience of education on emerging infectious diseases response^b^(Yes)	9.983	2.809	.194	3.554	.000
Willingness to Respond to a Disaster	1.964	.415	.267	4.731	.000
Preventive Behavior	1.153	.241	.256	4.790	.000
Job Satisfaction	1.558	.596	.148	2.615	.010

R^2^ = .348, Adjusted R^2^ = .332, F = 20.841, *p* < 0.001

Referent groups of dummy variables were ^a^Education (Associate’s degree), ^b^Experience of education on emerging infectious diseases response (No)

## Discussion

This study investigated the disaster competencies of public health nurses in the context of emerging infectious diseases, and analyzed the influencing factors of their disaster competencies based on the self-determination theory of Deci and Ryan. The major results of this study are discussed as follows.

A total of 32.23%, 17.36%, and 32.23% of the participants in this study belonged to the depression group, the anxiety group, and the stress group, respectively. These figures are significantly higher than 17.1%, 10%, and 12% of depression, anxiety, and stress prevalence in a study [35] conducted among the general population during the COVID-19 pandemic. These results support the findings of a study [36] conducted among nurses during the COVID-19 pandemic, which reported that stress, anxiety, and depression are significant problems. Furthermore, healthcare professionals are prone to experiencing increased depression, anxiety, and stress [35], which is related to the findings of previous studies [8, 9] that reported that increased patient contact heightens their risk of infection, which in turn causes stress. Considering the psychological impacts on public health nurses who are on the front line against COVID-19, active intervention is needed to minimize infection risk, provide mental support, and strengthen their coping skills. Group psychological interventions for managing stress will be helpful in addition to relevant activities, psychological intervention medical teams to care for common psychological problems, and a psychological assistance hotline [37].

In this study, the average score of burnout was 53.48 out of 95 points, which can be extrapolated to 56.29 out of 100 points. This figure is higher than 49.67 (out of 100 points) found in a study [38] that evaluated the level of burnout among nurses at hospitals prior to the COVID-19 breakout. It is higher than the figure of 50 (out of 100 points) recorded in a study [39] conducted among doctors from emergency medicine, acute medicine, general surgery, and trauma at a major trauma center, to evaluate the burnout level of general surgery doctors. The score is also higher than 49.2 (out of 100 points), which was measured in a study [40] conducted among nurses and doctors working in the emergency wards at hospitals during the COVID-19 pandemic using the same instrument as this study. Such a result signifies that the severity of burnout in public health nurses, who conduct epidemiological investigations, isolate infected patients, and operate screening clinics in local communities, is just as high as nurses and doctors working in hospitals to directly provide treatment to patients infected with COVID-19. Therefore, a protective and supportive work environment must be established to prevent prolonged working hours, provide rehabilitation and counseling services, and assure compensation in case of burnout to protect healthcare professionals including public health nurses and the overall healthcare system [10].

In this study, the average score of disaster competencies was 84.08 out of 168 points, which can be extrapolated to 50.05 out of 100 points. This figure is slightly lower than 55.95 (out of 100 points) found in a study [11] conducted among public health nurses and hospital nurses using the same instrument as in this study. The score of the prevention (pre-disaster) stage was the highest, followed by the mitigation (response) and the recovery (post-disaster) stages, which correspond to the findings of a previous study [11]. In particular, the low score of the recovery stage, which encompasses psychological interventions, corresponds to how psychological issues ranked the lowest mean familiarity score in previous studies [41–43] that evaluated the emergency preparedness of nurses. More than half of the disaster education programs that have been conducted among nurses over the last 20 years focused on contents related to the preparation and responses phases in the disaster cycle, and rarely covered the recovery phase because the role of nurses in the preparedness and response stages is extremely significant [44]. However, due to the nature of disasters, individuals and local communities may take a long time to recover from the damage caused by them [45, 46]. As nurses are critical healthcare professionals who serve various important roles in all the disaster stages: from the initial response to psychological healthcare in the recovery phase [3, 4], education programs that emphasize the importance of the recovery stage must be developed.

The study results showed that anxiety, burnout, job satisfaction, disaster competencies, and preventive behavior had a statistically significant difference according to age. The levels of anxiety and burnout were higher in younger participants, whereas job satisfaction, disaster competencies, and preventive behavior were higher in older participants. As the age and experience of nurses increase, confidence and job satisfaction also increase and the concomitant mental flexibility, acquired by adapting to the organizational environment, can lead to the improvement in resilience [38]. Additionally, resilience had a negative correlation with anxiety and depression in a study [47] conducting among the nurses in a COVID-19 unit and a non-COVID-19 unit. In a systematic review [48] on the resilience of nurses, resilience had a negative association with stress and burnout, but a positive relation with job satisfaction. Sense of coherence (SOC) is a similar concept as resilience; SOC strengthens resilience by allowing a stressful situation to be perceived as manageable and comprehensible through efficient use of resources [49]. It prevents post-traumatic stress in healthcare professionals [50], and a high level of SOC has been reported to be related to lower symptoms of anxiety, depression, distress, and mental overburden [12]. Resilience-building training that can alleviate anxiety and burnout in public health nurses is required considering how resilience stimulates acceptance and achievement of responsibility in nurses [51] and improves psychological and physical health conditions including burnout [52]. These resilience-building interventions should focus on improving external resources, including organizational support, in addition to strengthening internal resources such as self-management through a multifaceted approach [52]. Additionally, primarily deploying public health nurses with greater experience and skills on the front line must be considered.

The major variable of the study, disaster competencies, exhibited a positive correlation with age and job satisfaction, but a negative correlation with depression, stress, and burnout. This result is similar to the findings of a study [53] that reported that clinical competence has a significantly positive correlation with job satisfaction. Moreover, it is also in line with the results of a study [54] that reported that intercultural competence of nurses has a significant inverse relationship with perceived stress. Contrastingly, knowledge did not have a positive correlation with any of the variables, which differs from the results of previous studies [55–57] on emerging infectious diseases conducted among nurses, in which a correlation between knowledge and practice was observed. It is attributable to using the COVID-19 knowledge instrument of previous studies conducted among hospital nurses for the public health nurses in this study. Accordingly, an instrument of COVID-19 knowledge that is specialized for tasks and responsibilities of public health nurses must be developed.

Another result of this study that requires attention is that the levels of depression, anxiety, stress, and burnout were significantly higher among the participants with a permanent contract compared to those with a non-fixed-term or fixed-term contracts. This result is similar to the findings of a previous study [14] on emotional labor and job stress among public health nurses, which reported that the intensity of emotional labor and job stress of nurses with a permanent contract were higher than those of nurses with time-selective term, non-fixed term, or fixed-term contracts. It can be inferred that public health nurses with a permanent contract are mostly mid-level managers who face a high level of job stress because they must fulfill their administrative roles and responsibilities in addition to their regular work [58]. Public health nurses with a permanent contract also have a lower level of compassion satisfaction [14], which increases the quality of life by reducing burnout and enables individuals to continue performing given tasks under high stress, than the nurses with a non-permanent contract [59]. Therefore, the organizational climate must be improved to reduce the burden on permanent public health nurses by distributing or clearly defining the limit of their roles and responsibilities [14].

The study results showed that willingness to respond to a disaster has the greatest influence on disaster competencies. This result is in line with a previous study [7] conducted among hospital nurses, which reported that the “willingness to assume risk of involvement in a bioterrorism event” item of the motivation scale had the greatest influence on perceived competence in disaster preparedness. In a systematic review study [60] conducted among healthcare workers during an influenza pandemic, perceived personal safety, awareness of pandemic risk, clinical knowledge of the influenza pandemic, role-specific knowledge, pandemic response training, confidence in personal skills, and childcare obligations had a significant influence on willingness to work. Accordingly, systematic approaches, such as creating safe work conditions [61], high-quality training and education on pandemics [60], and support for caring for young children [62] are needed.

The experience of receiving education on emerging infectious diseases response was an influencing factor for disaster competencies in this study, which supports the findings of a previous study [63, 64] that reported that disaster-related training is an effective method for improving disaster-related knowledge and skills in nurses. Therefore, appropriate disaster education and training, such as realistic disaster exercises, mock drills, and disaster simulations must be provided periodically in order to improve the disaster competencies of public health nurses [65]. Unlike natural disasters such as typhoon and flood and man-made disasters such as fire and building collapse, special competencies such as those related to surveillance, infection control, quarantine monitoring, epidemiology, and immunization are required for pandemic response [66]. In this regard, public health nurses need to do the following tasks: taking samples and swabs, contact tracing, managing the hotlines, infection control training to the public, giving advice on infection control practices, following up on concerns expressed by the public, monitoring contacts at home quarantine, appropriate support provided to people confined to their homes, and so on [67]. In particular, when the epidemic of emerging infectious diseases such as COVID-19 is prolonged, new information continuously generated about the disease, such as virus mutation, epidemic pattern, vaccine development, and dissemination should be updated as soon as it is available. In addition, attention should be paid to changes in government quarantine measures caused by this new information. Furthermore, due to the prolonged epidemic of emerging infectious diseases, responders may experience psychological distress such as emotional exhaustion and burnout [68], so it is meaningful for educators responsible for strengthening disaster competencies to teach them how to take care of themselves and manage their own psychological wellbeing.

This study has the following limitations. First, caution is needed when generalizing the results of this study as only the public health nurses in the Busan-Gyeongnam region were recruited. Second, caution is needed when interpreting its results as the reliability of the COVID-19 knowledge instrument, which targeted hospital nurses in previous studies, was fairly low for this study (Cronbach’s alpha of 0.378) because the instrument was partially revised and supplemented. Third, all variables were measured at one point in time rather than in a longitudinal observation because of the cross-sectional design. Therefore, the significant association between disaster competencies and variables may not imply a causal relationship. Fourth, response bias may affect the results because the survey was conducted in a self-report format; in particular, the survey cannot replace clinical diagnostic interviews for psychological variables. Nonetheless, this study has significance in that it investigated disaster competencies and important their influencing factors based on Deci and Ryan’s self-determination theory, even when there is insufficient research on public health nurses, despite their critical role in responding to emerging infectious diseases.

## Conclusions

This descriptive cross-sectional study investigated disaster competencies of public health nurses in the context of emerging infectious diseases, and the influencing factors of their disaster competencies based on the self-determination theory of Deci and Ryan. According to the results of the multiple regression analysis, the influencing factors for disaster competencies of public health nurses include willingness to respond to a disaster, preventive behavior, experience of receiving education on emerging infectious diseases response, public health center experience, job satisfaction, and educational level. More specifically, disaster competencies of public health nurses were higher when the participants’ willingness to respond to a disaster was higher, they had better preventive behavior, more public health center experience, and higher job satisfaction, or the participants possessed or were pursuing a master’s degree in nursing. Willingness to respond to a disaster was found to have the greatest influence on disaster competencies.

The following proposals are based on the limitations and results of this study. First, a study should be conducted to develop a COVID-19 knowledge instrument that adequately reflects the competencies and range of tasks of public health nurses. Second, an intervention study should be conducted to improve the disaster competencies and the psychological well-being of public health nurses. Third, the organizational climate should be improved through a protective and supportive work environment, primarily deploying more experienced and better skilled nurses on the front line, and reducing the responsibilities and tasks of permanent public health nurses.

## Supplementary Information

**Additional file 1:** **Table A. 1.** Differences in Variables according to Sociodemographic Characteristics (N=242).

## Data Availability

There is no public access to all data generated or analyzed during this study to preserve the privacy of the identities of the individuals. The dataset that supports the conclusions is available to the corresponding author upon request.

## Abbreviations

COVID-19: Coronavirus disease 2019
PHQ: Patient health questionnaire
GAD: Generalized Anxiety Disorder
COPSOQ: Copenhagen Psychosocial Questionnaire
BEPSI: Brief Encounter Psychosocial Instrument
DPET: Disaster Preparedness Evaluation Tool
CVI: Content validity index
KR-20: Kuder-Richardson 20
IRB: Institutional Review Board
SOC: Sense of coherence
