# Supplementary material for: A cross-sectional study on public health nurses' disaster competencies and influencing factors during the COVID-19 pandemic in Korea
Source: BMC Public Health. 2022 Apr 13;22:731. doi: 10.1186/s12889-022-13091-2 (PMC9005315; doi:10.1186/s12889-022-13091-2)
Supplement: Supplementary file 1 — Additional file 1: Table A. 1. Differences in Variables according to Sociodemographic Characteristics (N=242). [file 12889_2022_13091_MOESM1_ESM.doc]

**Additional File**

**Table A. 1. Differences in Variables according to Sociodemographic Characteristics (N=242)**

| **Characteristics** | **Categories** | **Depression** | | **Anxiety** | | **Job Satisfaction** | | **Stress** | | **Burn Out** | |
| --- | --- | --- | --- | --- | --- | --- | --- | --- | --- | --- | --- |
|  |  | **M±SD** | **t/F(p)** | **M±SD** | **t/F(p)** | **M±SD** | **t/F(p)** | **M±SD** | **t/F(p)** | **M±SD** | **t/F(p)** |
| Gender | Male | 5.83±  3.37 | -0.361  (0.718) | 4.33±  3.14 | -0.030  (0.976) | 8.50±  1.87 | -1.689  (0.091) | 2.00±  0.85 | -1.118  (0.264) | 52.50±12.53 | -0.080  (0.936) |
|  | Female | 7.55±  5.71 |  | 5.13±  4.97 |  | 9.95±  2.34 |  | 2.15±  0.61 |  | 53.50±16.26 |  |
| Age | ≦29 | 8.02±  5.67 | 7.061  (0.133) | 4.98±  4.75 | 10.503  (0.033) | 9.84±  2.37 | 21.262  (0.000) | 2.15±  0.63 | 7.709  (0.103) | 56.14±16.80 | 35.250  (0.000) |
|  | 30-39 | 8.20±  5.91 |  | 6.43±  5.59 |  | 9.22±  2.29 |  | 2.23±  0.62 |  | 58.54±15.86 |  |
|  | 40-49 | 6.73±  5.07 |  | 3.80±  3.84 |  | 10.51±2.09 |  | 2.04±  0.66 |  | 49.39±14.62 |  |
|  | 50-59 | 6.12±  5.79 |  | 3.85±  4.18 |  | 11.06±2.15 |  | 2.03±  0.48 |  | 42.00±9.72 |  |
|  | ≧60 | 4.33±  3.51 |  | 3.67±  3.06 |  | 10.00±2.65 |  | 2.20±  0.40 |  | 38.67±8.74 |  |
| Education | AD | 6.69±  6.03 | 3.450  (0.178) | 4.48±  4.47 | 0.862  (0.650) | 10.98±1.96 | 16.555  (0.000) | 2.06±  0.65 | 4.017  (0.134) | 47.33±14.58 | 11.110  (0.004) |
|  | BD | 7.84±  5.62 |  | 5.27±  5.09 |  | 9.68±  2.38 |  | 2.15±  0.60 |  | 55.00±16.42 |  |
|  | MD | 6.50±  4.80 |  | 5.38±  4.70 |  | 9.00±  1.90 |  | 2.35±  0.66 |  | 56.88±13.89 |  |
| Religion | Christian | 6.78±  5.29 | 8.414  (0.078) | 4.42±  4.31 | 11.805  (0.019) | 9.93±  2.43 | 5.010  (0.286) | 2.15±  0.53 | 5.181  (0.269) | 50.90±15.78 | 6.309  (0.177) |
|  | Catholic | 9.74±  6.07 |  | 7.30±  5.21 |  | 9.22±  2.22 |  | 2.37±  0.70 |  | 59.13±13.39 |  |
|  | Buddhist | 8.07±  5.99 |  | 5.40±  4.74 |  | 9.96±  2.17 |  | 2.15±  0.46 |  | 53.47±16.35 |  |
|  | No Religion | 7.30±  5.60 |  | 4.98±  5.10 |  | 9.98±  2.40 |  | 2.11±  0.66 |  | 53.63±16.76 |  |
|  | Others | 3.40±  0.89 |  | 1.20±  1.30 |  | 11.20±1.30 |  | 1.84±  0.74 |  | 44.20±5.54 |  |
| Marital Status | Single | 7.60±  5.52 | 0.503  (0.778) | 5.07±  4.80 | 1.555  (0.460) | 9.55±  2.34 | 10.463  (0.005) | 2.12±  0.58 | 1.879  (0.391) | 56.35±16.45 | 7.370  (0.025) |
|  | Married | 7.32±  5.60 |  | 5.02±  4.97 |  | 10.27±2.30 |  | 2.15±  0.65 |  | 50.90±15.50 |  |
|  | Other | 11.25±11.00 |  | 9.00±  6.98 |  | 8.25±  0.96 |  | 2.45±  0.44 |  | 61.00±18.51 |  |
| Child | One or more | 6.79±  5.41 | -1.680  (0.094) | 4.55±  4.44 | -1.508  (0.123) | 10.44±2.03 | 3.126  (0.002) | 2.12±  0.59 | -0.545  (0.586) | 48.82±14.18 | -4.039  (0.000) |
|  | None | 8.03±  5.81 |  | 5.51±  5.24 |  | 9.54±  2.47 |  | 2.16±  0.64 |  | 56.86±16.70 |  |
| First Child’s Age | ≦5 | 6.86±  5.29 | 3.369  (0.338) | 5.71±  5.24 | 3.823  (0.281) | 10.86±2.28 | 4.748  (0.191) | 2.26±  0.53 | 6.619  (0.085) | 56.50±16.46 | 12.847  (0.005) |
|  | 6-12 | 8.06±  5.78 |  | 5.26±  4.87 |  | 10.12±1.87 |  | 2.24±  0.76 |  | 52.21±13.97 |  |
|  | 13-18 | 5.33±  3.52 |  | 4.13±  3.80 |  | 10.07±1.83 |  | 1.95±  0.44 |  | 48.27±13.55 |  |
|  | ≧19 | 6.13±  5.55 |  | 3.62±  3.90 |  | 10.79±2.13 |  | 2.02±  0.44 |  | 43.00±11.39 |  |
| TWE | <1 | 9.83±  5.95 | 7.211  (0.205) | 7.00±  6.47 | 8.880  (0.114) | 9.92±  3.03 | 15.938  (0.007) | 2.37±  0.76 | 4.291  (0.508) | 56.33±19.03 | 18.147  (0.003) |
|  | 1-4 | 8.16±  5.62 |  | 4.80±  4.86 |  | 9.79±  2.26 |  | 2.10±  0.57 |  | 55.45±15.90 |  |
|  | 5-9 | 7.78±  5.76 |  | 6.19±  5.32 |  | 9.40±  2.46 |  | 2.21±  0.61 |  | 57.32±16.52 |  |
|  | 10-19 | 6.63±  5.35 |  | 4.51±  4.47 |  | 9.94±  2.24 |  | 2.12±  0.65 |  | 51.93±15.89 |  |
|  | 20-29 | 6.37±  4.46 |  | 2.95±  2.61 |  | 10.95±1.75 |  | 2.04±  0.54 |  | 45.68±11.60 |  |
|  | ≧30 | 7.27±  7.54 |  | 4.87±  5.15 |  | 11.53±1.46 |  | 2.04±  0.59 |  | 41.93±10.66 |  |
| PHNE | <1 | 6.92±  5.02 | 2.021  (0.846) | 5.08±  5.01 | 2.930  (0.711) | 10.45±2.33 | 13.111  (0.022) | 2.04±  0.54 | 1.932  (0.858) | 51.17±15.66 | 12.316  (0.031) |
|  | 1-4 | 8.02±  6.11 |  | 5.19±  5.37 |  | 9.71±  2.43 |  | 2.18±  0.68 |  | 54.77±16.16 |  |
|  | 5-9 | 7.63±  5.43 |  | 5.90±  4.91 |  | 9.42±  2.28 |  | 2.16±  0.55 |  | 58.35±17.35 |  |
|  | 10-19 | 6.81±  4.68 |  | 4.00±  3.48 |  | 9.89±  2.18 |  | 2.15±  0.64 |  | 50.36±15.17 |  |
|  | 20-29 | 7.83±  7.78 |  | 5.00±  4.90 |  | 10.83±2.14 |  | 2.13±  0.69 |  | 47.67±14.25 |  |
|  | ≧30 | 6.55±  7.03 |  | 4.73±  4.90 |  | 11.64±1.21 |  | 2.04±  0.52 |  | 42.00±6.99 |  |
| Employment type | Permanent | 8.46±  5.97 | 9.418  (0.000) | 5.99±  5.22 | 10.844  (0.000) | 9.53±  2.29 | 9.595  (0.000) | 2.21±  0.66 | 3.700  (0.026) | 57.38±16.26 | 21.154  (0.000) |
|  | Non-fixed term | 5.34±  3.98 |  | 2.91±  3.34 |  | 10.56±1.93 |  | 1.93±  0.44 |  | 44.09±10.82 |  |
|  | Fixed-term | 4.83±  3.84 |  | 2.78±  2.97 |  | 11.19±2.35 |  | 2.02±  0.44 |  | 42.94±10.78 |  |
| Work Location | Busan | 7.59±  5.55 | 0.260  (0.795) | 5.03±  4.78 | -0.288  (0.774) | 9.84±  2.34 | -0.585  (0.559) | 2.19±  0.58 | 1.466  (0.144) | 54.19±16.09 | 0.796  (0.427) |
|  | Gyeongnam | 7.40±  5.84 |  | 5.21±  5.15 |  | 10.02±2.34 |  | 2.08±  0.66 |  | 52.51±16.28 |  |
| Place of Employment | PHC | 7.71±  5.76 | 6.361  (0.273) | 5.22±  5.01 | 6.317  (0.277) | 9.78±  2.27 | 10.206  (0.070) | 2.15±  0.59 | 1.419  (0.922) | 54.26±±16.30 | 5.051  (0.410) |
|  | HCB | 5.00±  3.52 |  | 3.00±  3.41 |  | 9.67±  2.34 |  | 2.17±  0.87 |  | 47.50±10.41 |  |
|  | CHC | 7.20±  3.96 |  | 5.00±  3.08 |  | 12.06±2.51 |  | 2.16±  0.48 |  | 53.40±13.69 |  |
|  | DSC | 4.00±  2.83 |  | 2.00±  1.73 |  | 10.29±2.93 |  | 1.89±  0.58 |  | 45.71±8.58 |  |
|  | CHPC | 9.86±  7.38 |  | 7.71±  6.68 |  | 10.00±2.89 |  | 2.34±  1.24 |  | 51.29±21.84 |  |
|  | Others | 5.38±  4.34 |  | 4.13±  3.76 |  | 11.62±1.77 |  | 2.08±  0.53 |  | 46.13±16.17 |  |
| Work Department | IDR | 8.94±  5.87 | 1.793  (0.074) | 6.38±  5.53 | 1.938  (0.054) | 9.50±  2.41 | -1.026  (0.306) | 2.18±  0.66 | 0.428  (0.669) | 59.85±17.17 | 2.900  (0.004) |
|  | NIDR | 7.30±  5.68 |  | 4.84±  4.78 |  | 9.87±  2.22 |  | 2.14±  0.57 |  | 52.41±15.62 |  |
| EID | Yes | 7.06±  5.50 | -1.699  (0.091) | 4.40±  4.51 | -2.989  (0.003) | 10.18±2.27 | 2.411  (0.017) | 2.10±  0.544 | -1.573  (0.117) | 50.90±15.29 | -3.513  (0.001) |
|  | No | 8.36±  5.91 |  | 6.47±  5.41 |  | 9.42±  2.38 |  | 2.23±  0.73 |  | 58.41±16.71 |  |
| EWCSC | Yes | 7.76±  5.78 | -1.916  (0.055) | 5.31±  5.04 | -1.746  (0.081) | 9.76±  2.27 | -2.883  (0.004) | 2.17±  0.62 | -1.870  (0.061) | 54.53±16.33 | -3.135  (0.002) |
|  | No | 5.21±  3.81 |  | 3.29±  3.24 |  | 11.38±2.46 |  | 1.89±  0.52 |  | 43.88±10.60 |  |
| Total | Mean±SD | 7.51±  5.67 |  | 5.11±  4.93 |  | 9.92±  2.33 |  | 2.14±  0.62 |  | 53.48±16.16 |  |
|  | Range | 0-24 |  | 0-21 |  | 4-16 |  | 1-5 |  | 19-95 |  |

**Note:** AD: Associate’s degree; BD: Bachelor’s degree; MD: Pursuing/Master’s degree; TWE: Total Work Experience as a Nurse; PHNE: Public Health Nurse Experience; EID: Education for infectious diseases; EWCSC: Experience of Working at COVID-19 Screening Clinics; PHC: Public health center; HCB: Health center branch; CHC: Community health center; DSC: District service center; CHPC: Community health promotion center; IDR: Infectious disease related; NIDR: Non-infectious disease related

**Table A1.** Cont.

| **Characteristics** | **Categories** | **Disaster Competencies** | | **Knowledge** | | **Preventive Behavior** | | **Willingness** | |
| --- | --- | --- | --- | --- | --- | --- | --- | --- | --- |
|  |  | **M±SD** | **t/F(p)** | **M±SD** | **t/F(p)** | **M±SD** | **t/F(p)** | **M±SD** | **t/F(p)** |
| Gender | Male | 84.50±42.34 | -0.018  (0.986) | 12.83±1.94 | -1.484  (0.138) | 46.67±7.09 | -0.405  (0.685) | 3.67±  4.03 | -0.219  (0.826) |
|  | Female | 84.07±24.28 |  | 14.11±2.15 |  | 45.79±5.41 |  | 3.71±  3.33 |  |
| Age | ≦29 | 83.20±24.59 | 14.793  (0.005) | 13.94±2.19 | 1.420  (0.841) | 45.03±5.51 | 10.468  (0.033) | 3.97±  3.21 | 8.998  (0.061) |
|  | 30-39 | 78.23±21.49 |  | 14.02±2.26 |  | 45.25±5.78 |  | 2.96±  3.37 |  |
|  | 40-49 | 88.22±26.23 |  | 14.16±2.02 |  | 46.65±4.85 |  | 4.02±  3.39 |  |
|  | 50-59 | 94.18±28.01 |  | 14.39±1.95 |  | 47.79±4.77 |  | 4.76±  3.19 |  |
|  | ≧60 | 98.67±11.85 |  | 14.33±3.51 |  | 43.33±5.69 |  | 4.00±  3.61 |  |
| Education | AD | 90.21±24.10 | 10.077  (0.006) | 14.23±2.00 | 0.213  (0.899) | 46.15±5.63 | 1.084  (0.582) | 4.56±  3.28 | 5.585  (0.061) |
|  | BD | 81.07±24.04 |  | 14.02±2.20 |  | 45.67±5.43 |  | 3.45±  3.32 |  |
|  | MD | 96.81±28.01 |  | 14.25±2.18 |  | 46.25±5.26 |  | 3.69±  3.54 |  |
| Religion | Christian | 85.05±24.87 | 0.291  (0.990) | 13.65±1.83 | 3.664  (0.453) | 46.70±5.24 | 3.280  (0.512) | 4.03±  3.50 | 3.202  (0.525) |
|  | Catholic | 83.87±22.39 |  | 13.61±2.64 |  | 45.57±6.16 |  | 4.57±  3.27 |  |
|  | Buddhist | 85.53±27.90 |  | 14.27±2.07 |  | 45.73±5.61 |  | 3.47±  3.50 |  |
|  | No Religion | 83.19±24.12 |  | 14.22±2.17 |  | 45.48±5.34 |  | 3.61±  3.25 |  |
|  | Others | 87.20±28.87 |  | 14.60±2.30 |  | 49.00±4.85 |  | 1.80±  3.49 |  |
| Marital Status | Single | 80.85±24.06 | 4.247  (0.120) | 14.06±2.22 | 2.836  (0.242) | 45.07±5.81 | 5.104  (0.078) | 3.54±  3.31 | 5.193  (0.075) |
|  | Married | 86.89±24.88 |  | 14.16±2.08 |  | 46.42±5.12 |  | 3.95±  3.37 |  |
|  | Other | 78.25±33.27 |  | 12.25±2.63 |  | 45.75±3.87 |  | 0.25±  0.50 |  |
| Child | One or more | 88.42±25.98 | 2.353  (0.019) | 14.18±2.02 | 0.578  (0.564) | 46.53±5.11 | 1.763  (0.079) | 3.98±  3.28 | 1.088  (0.278) |
|  | None | 80.91±23.38 |  | 14.01±2.25 |  | 45.29±5.63 |  | 3.51±  3.39 |  |
| First Child’s Age | ≦5 | 83.79±29.53 | 2.504  (0.475) | 13.43±1.99 | 2.789  (0.425) | 45.64±4.67 | 8.640  (0.034) | 2.64±  3.10 | 9.273  (0.026) |
|  | 6-12 | 85.82±24.29 |  | 14.06±2.06 |  | 45.41±5.13 |  | 3.18±  3.28 |  |
|  | 13-18 | 91.93±23.49 |  | 13.87±2.45 |  | 49.73±4.57 |  | 4.00±  3.25 |  |
|  | ≧19 | 92.95±28.04 |  | 14.56±1.85 |  | 46.31±5.02 |  | 5.15±  3.06 |  |
| TWE | <1 | 79.00±23.81 | 18.585  (0.002) | 12.83±2.33 | 5.471  (0.361) | 46.17±5.42 | 2.993  (0.701) | 3.33±  3.00 | 7.035  (0.218) |
|  | 1-4 | 81.57±23.68 |  | 14.25±2.25 |  | 45.05±6.05 |  | 3.61±  3.33 |  |
|  | 5-9 | 82.10±22.09 |  | 14.12±2.31 |  | 45.60±5.05 |  | 3.19±  3.24 |  |
|  | 10-19 | 80.55±26.62 |  | 14.00±1.88 |  | 46.24±5.64 |  | 3.88±  3.54 |  |
|  | 20-29 | 99.11±23.17 |  | 13.95±1.81 |  | 46.32±4.74 |  | 4.37±  3.40 |  |
|  | ≧30 | 103.87±22.16 |  | 14.80±2.31 |  | 46.80±5.28 |  | 5.27±  3.04 |  |
| PHNE | <1 | 84.45±25.92 | 18.538  (0.002) | 13.67±2.14 | 7.316  (0.198) | 44.13±6.06 | 7.329  (0.197) | 3.77±  3.19 | 3.599  (0.608) |
|  | 1-4 | 80.55±22.84 |  | 14.41±2.24 |  | 45.92±5.43 |  | 3.65±  3.30 |  |
|  | 5-9 | 81.69±24.71 |  | 13.71±2.06 |  | 46.08±5.11 |  | 3.27±  3.49 |  |
|  | 10-19 | 84.97±25.06 |  | 13.83±1.84 |  | 46.17±5.38 |  | 3.92±  3.55 |  |
|  | 20-29 | 112.67±23.47 |  | 14.33±2.25 |  | 48.33±3.50 |  | 3.83±  3.37 |  |
|  | ≧30 | 107.00±18.54 |  | 14.91±2.43 |  | 47.18±5.21 |  | 5.09±  3.27 |  |
| Employment type | Permanent | 83.81±24.99 | 1.174(0.311) | 14.13±2.19 | 0.154(0.857) | 45.25±5.18 | 3.716(0.026) | 3.60±  3.37 | 0.439  (0.645) |
|  | Non-fixed term | 79.97±21.61 |  | 13.91±2.13 |  | 47.81±6.88 |  | 3.78±  3.43 |  |
|  | Fixed-term | 89.03±25.94 |  | 14.03±2.05 |  | 46.75±4.84 |  | 4.17±  3.17 |  |
| Work Location | Busan | 81.55±25.31 | -1.859  (0.064) | 14.22±2.10 | 1.180  (0.239) | 45.94±4.98 | 0.427  (0.670) | 3.81±  3.35 | 0.535  (0.593) |
|  | Gyeongnam | 87.50±23.63 |  | 13.89±2.22 |  | 45.63±6.04 |  | 3.57±  3.35 |  |
| Place of Employment | PHC | 83.30±24.64 | 3.981  (0.552) | 14.08±2.17 | 4.111  (0.534) | 45.61±5.53 | 4.185  (0.523) | 3.62±  3.33 | 4.669  (0.458) |
|  | HCB | 98.33±30.92 |  | 12.67±1.37 |  | 47.67±3.50 |  | 3.83±  4.22 |  |
|  | CHC | 92.80±25.79 |  | 14.40±1.14 |  | 47.60±7.40 |  | 4.20±  3.56 |  |
|  | DSC | 92.29±27.10 |  | 15.00±2.52 |  | 45.71±6.37 |  | 5.00±  3.27 |  |
|  | CHPC | 72.71±21.88 |  | 14.00±2.94 |  | 47.14±4.06 |  | 2.57±  3.36 |  |
|  | Others | 91.00±20.75 |  | 14.38±1.30 |  | 47.38±3.54 |  | 5.50±  3.12 |  |
| Work Department | IDR | 83.06±25.95 | -0.082  (0.935) | 14.12±2.04 | 0.148  (0.882) | 45.12±4.63 | -0.748  (0.445) | 3.50±  3.32 | -0.292  (0.770) |
|  | NIDR | 83.38±24.27 |  | 14.06±2.22 |  | 45.78±5.80 |  | 3.66±  3.34 |  |
| EID | Yes | 89.54±23.58 | 4.984  (0.000) | 14.21±2.09 | 1.251  (0.212) | 46.18±5.71 | 1.477  (0.141) | 4.18±  3.34 | 3.076  (0.002) |
|  | No | 73.61±23.63 |  | 13.84±2.26 |  | 45.10±4.84 |  | 2.81±  3.18 |  |
| EWCSC | Yes | 83.53±24.38 | -0.854  (0.393) | 14.09±2.14 | -0.240  (0.810) | 45.70±5.24 | -1.114  (0.265) | 3.66±  3.32 | -0.931  (0.352) |
|  | No | 89.04±27.88 |  | 14.00±2.28 |  | 46.83±7.10 |  | 4.17±  3.61 |  |
| Total | Mean±SD | 84.08±24.74 |  | 14.08±2.15 |  | 45.81±5.44 |  | 3.71±  3.34 |  |
|  | Range | 28-168 |  | 0-20 |  | 14-56 |  | 0-8 |  |

**Note:** AD: Associate’s degree; BD: Bachelor’s degree; MD: Pursuing/Master’s degree; TWE: Total Work Experience as a Nurse; PHNE: Public Health Nurse Experience; EID: Education for infectious diseases; EWCSC: Experience of Working at COVID-19 Screening Clinics; PHC: Public health center; HCB: Health center branch; CHC: Community health center; DSC: District service center; CHPC: Community health promotion center; IDR: Infectious disease related; NIDR: Non-infectious disease related
